# Effect of Metabolite Extract of *Streptomyces hygroscopicus* subsp. *hygroscopicus* on *Plasmodium falciparum* 3D7 in Vitro

**Published:** 2019

**Authors:** Loeki Enggar FITRI, Annisa ALKARIMAH, Alfian Wika CAHYONO, Wahyudha Ngatiril LADY, Agustina Tri ENDHARTI, Rivo Yudhinata Brian NUGRAHA

**Affiliations:** 1. Department of Parasitology, Malaria Research Group, Faculty of Medicine, Universitas Brawijaya, Malang, Indonesia; 2. Master Program in Biomedical Science, Faculty of Medicine, Universitas Brawijaya, Malang, Indonesia; 3. Labarotory of Biomedic, Faculty of Medicine, Universitas Brawijaya, Malang, Indonesia

**Keywords:** *Streptomyces hygroscopicus* subsp hygroscopicus, *Plasmodium falciparum* 3D7, DNA parasite density

## Abstract

**Background:**

Malaria eradication has been complicated by the repeated emergence of antimalarial drug resistances. We aimed to determine whether a metabolite extract of *Streptomyces hygrocopicus* subsp. *hygroscopicus* could decrease the viability of *Plasmodium falciparum* 3D7 in vitro.

**Methods:**

*S. hygroscopicus* subsp. *hygroscopicus* isolates were inoculated and fermented on the ISP4 medium. The fermented *S. hygroscopicus* was mixed with ethylacetate 1:5 (v/v), and the solvent phase was evaporated. Several concentrations of isolated extract was added to the *P. falciparum* 3D7 culture containing trophozoite and schizont stages in 24 wells plates when the degree of parasite-infected erythrocytes reached 5%, then incubated for 8 hours. DNA parasite density was measured using flow cytometry, parasite degree and morphology were observed under microscopic by Giemsa-stained smears.

**Results:**

The metabolite extract affected the morphology of almost all of parasite asexual stages. Schizonts and trophozoites failed to grow and appeared damaged with pycnotic cores and loss of cytoplasmic content. At 8 hours there was a significant decrease in DNA parasite density in culture exposed to 2.6 mg/ml and 13 mg/ml (*P* = 0.002; *P* = 0.024) of the extract. The degree of parasite-infected erythrocytes was decreased from the beginning of exposure (0.02 mg/ml of the extract). There was a significant inverse correlation between the concentration of extract and the degree of parasite-infected erythrocytes as well as the density of DNA parasite (r = −0.772, *P* = 0.000; r =−0.753; *P* =0.000).

**Conclusion:**

Metabolite extract of *S. hygroscopicus* subsp. *hygroscopicus* causes morphological damage, decreases the degree of parasite-infected erythrocytes and the DNA density of *P. falciparum* 3D7 in vitro.

## Introduction

The greatest threat to malaria control is antimalarial drug resistances. To date, three of the five species that cause malaria, *Plasmodium falciparum*, *P. vivax*, and *P. malariae* have been found to develop resistance (1). Drug resistance is complicated by the existence of cross-resistance occurring between drugs with similar mechanisms of action.

The ubiquitin - proteasome system (UPS) encompasses the process of post-translational modification of proteins in the regulation of eukaryotic cells (2). Through specific targeted destruction of regulatory proteins, this pathway participates in the regulation of numerous cellular and physiological functions including cell proliferation, cell death, and signal transduction. Control of proteins plays a significant role in the *Plasmodium* life cycle. *Plasmodium* proteins were depressed by the presence of heat due to fever in patients a process mediated by a polyubiquitination mechanism (3–5).

*Streptomyces hygroscopicus* is a Gram-positive soil bacterium that forms filaments. The entire genus *Streptomyces* has the ability to produce secondary metabolites that have antibiotic, antifungal, antiviral, antitumor, and immunosuppressive activity (6). *S. hygrocopicus* subsp hygroscopicus secondary metabolites contain eponemycin, a compound that has antitumor and antibiotic activity (7). Eponemycin, as one class of epoxyketone peptide, displays anti-tumor activity through inhibition of proteasome function and further disrupt the ubiquitin-proteasome system works. Inhibition of proteasome function causes changes in cell morphology and ends in apoptosis (8). The mechanism of action affecting the proteasome was later to become a target of anti-malarial therapy (9,10).

We aimed to determine whether the same metabolite extract can act as anti-malarial agent to *P. falciparum* in vitro.

## Materials and Methods

### Experimental Design

This study was an in vitro experimental study using *P. falciparum* 3D7 culture. Parasite culture was exposed by increasing doses of a metabolite extract of *S. hygrocopicus* subsp. hygroscopicus 0.02, 0.104, 0.52, 2.6, 13 mg/ml respectively. We observed its effect compared to control cell that did not expose to the extract. Each group was quadruplet in replication.

### Preparation of Bacterial Isolate Medium

One liter of medium ISP4 (International *Streptomyces* Project) was composed of 10 g soluble starch, 1g K_2_HPO_4_, 1 g MgSO_4_.7H_2_O, 1g NaCl, 2 g (NH_4_) 2SO_4_, 2 g CaCO_3_, 1 g trace salt solution (0.1 g Fe_2_SO_4_.7H_2_O, ZnSO_4_.7H_2_O, MnCl_2_.4H_2_O 0.1 g, distilled water 1 ml), 20 g agar, and one liter distilled water with pH 7.0 to 7.4 (11). The solution was autoclaved at 121°C for 15 min, and then *S. hygroscopicus* subsp. hygroscopicus isolates were inoculated and fermented on the medium at 28 °C shaking incubator (12).

### Preparation of *S. hygroscopicus* subsp. *hygroscopicus* Isolate

*S. hygrocopicus* subsp. hygroscopicus was obtained from the Microbiology Laboratory LIPI (LIPI-MC) Cibinong, Indonesia, and from this culture the metabolite-containing extract was produced and prepared as described before (13). Briefly the fermented *S. hygroscopicus* was mixed with ethylacetate 1:5 (v/v), shaken for 1 hour, and deposited in a separate funnel for 4 hours. After that, the water phase was discarded and the solvent phase (ethylacetate) was evaporated in a water-bath 80 – 90°C.

### Plasmodium falciparum 3D7 Culture and Exposure to Metabolite Extract

Asynchronous culture of *P. falciparum* 3D7 were grown in RPMI-1640 medium containing 10% human serum type O (10% complete medium) and 5% hematocrit by the method of Trager and Jensen (1976) (14). Crude metabolite containing extract was dissolved in DMSO (2 – 10%) with a sonicator. It was then diluted with RPMI and aliquoted in several concentration 0.02 mg/ml (A), 0.104 mg/ml (B), 0.52 mg/ml (C), 2.6 mg/ml (D) and 13 mg/ml (E) respectively. Extract was added to the in vitro parasite culture in 24 wells plates (Corning Inc.) when the degree of parasite-infected erythrocytes reached 5%. The degree of parasite and parasite morphology were observed after 8 hours' incubation at 37 °C with 5% CO_2_ (15, 16). They were determined under microscopic observation of 1,000 erythrocytes using 1000x magnification after Giemsa staining.

### Flow cytometry for DNA Parasite Density

DNA parasite density measurement using flow cytometry was done after 8 hours' exposure to crude extract. The total volume of each well (approximately 1 ml) was harvested and pelleted in a centrifuge. About 20 μL of erythrocyte pellets each well were washed using PBS twice. For cell fixation, pellets were re-suspended with 1 mL PBS containing 0.25% (v/v) glutaraldehyde then incubated in room temperature for 20 minutes. Cells were centrifuged 450xg for 5 minutes then washed pellets with PBS twice. Cells were re-suspended with 0.5 mL of PBS containing 0.01% saponin, incubated at room temperature for 5 minutes, then washed again with PBS twice. Subsequently, the pellet was re-suspended in 200 μL PBS containing 40 μL RNAse A (1 mg/ml), incubated at 37°C for 40–60 minutes, and washed again with PBS twice. Cells were re-suspended in 0.5 – 1.0 mL of PBS containing 2% FCS. Propidium Iodine (PI) solution (to a final concentration of 10 μg/mL) was added as a DNA dye for the *Plasmodium* cells. This suspension was incubated in dark room for 1–2 hours at 37 °C. It was then analyzed with a flow cytometer 488 nm laser for excitation and emission detected from intercalating PI in 670 nm long pass filtered FL3 channel. FACScan and FACScalibur from BD were used and emission detected in FL3 (14).

### Data Analysis

Parasite morphology was analyzed descriptively by comparing the treatment and control groups. The degree of parasite and DNA parasite density, were analyzed with SPSS 16 with one-way ANOVA test, post hoc test, Pearson correlation test, and linear regression test with *P* < 0.05.

## Results

### Parasite Morphology

**Fig. 1:**
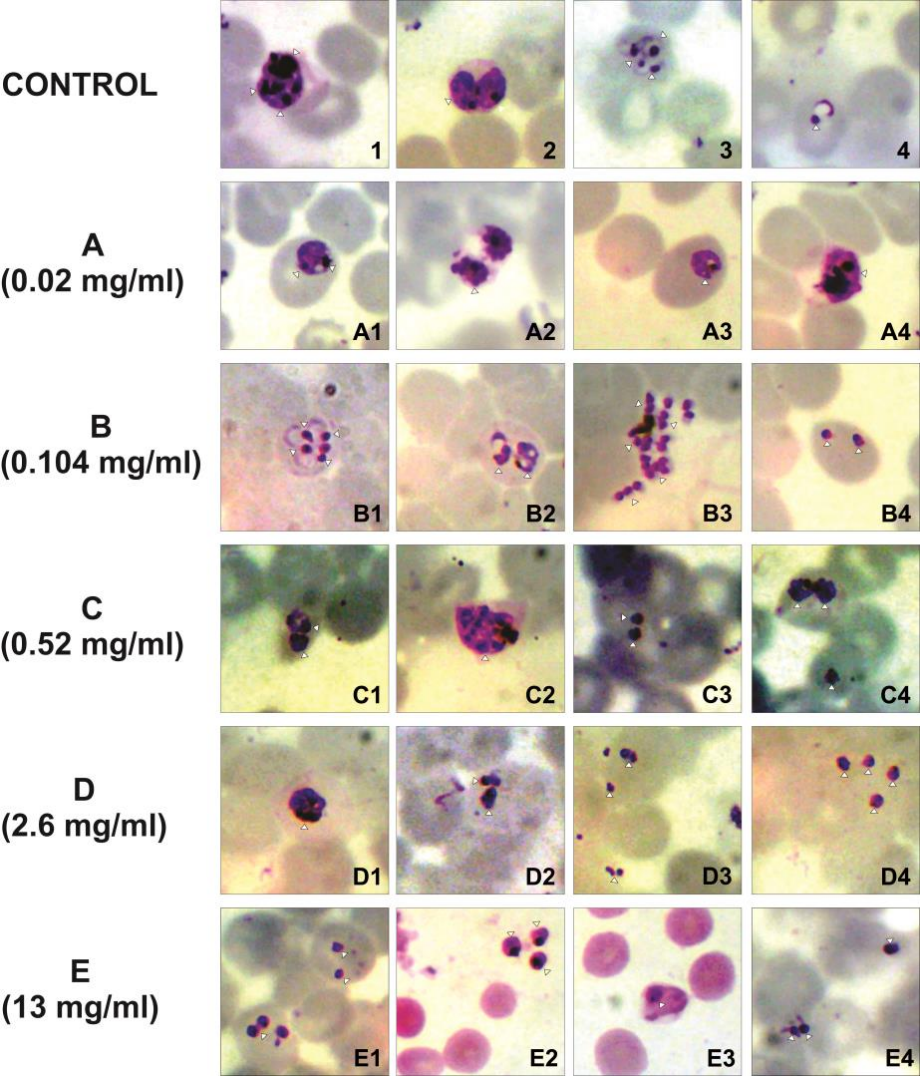
*Plasmodium falciparum* 3D7 morphology alteration after 8 hours *S. hygroscopicus* subsp. hygrocopicus crude metabolite extracts incubation. Giemsa-stained smears of asynchronized asexual blood stages consisted control and treatment group (A1 – 4, B1 – 4, C1 – 4, D1 – 4, and E1 – 4) that showed change of parasite morphology (white arrows). Majority of parasite morphology became crisis form in treatment groups. A= *S. hygroscopicus* subsp. hygrocopicus crude metabolite extracts 0.02 mg/ml, B= *S. hygroscopicus* subsp. hygrocopicus crude metabolite extracts 0.104 mg/ml, C= *S. hygroscopicus* subsp. hygrocopicus crude metabolite extracts 0.52 mg/ml, D= *S. hygroscopicus* subsp. hygrocopicus crude metabolite extracts 2.6 mg/ml and E= *S. hygroscopicus* subsp. hygrocopicus crude metabolite extracts 13 mg/ml

Parasite morphology was observed after 8 hours as shown in Fig. 1. Parasite morphologies in control group showed that schizont represented the predominant stage of the parasite in cultures (Fig. 1 – control 1–3). Mature trophozoites and ring-form stages also existed (Fig. 1 – control 4). Many immature merozoites were observed outside the erythrocytes. Exposure to 0.02 mg/ml of the extract already showed some double chromatin infections (Fig. 1 – A1, 2). Mature trophozoites and schizonts were also observed infrequently about 1 – 2 parasites per field of view (Fig. 1 – A3, 4). At the 0.104 mg/ml dose disturbances of schizogony development were evident as marked by thin cytoplasm (B1) and irregular ring-forms with damaged cytoplasm (B2). There were many thick and dark parasites with the pycnotic nuclei and loss of cytoplasmic volume (B4). This condition is characteristic of crisis forms. Merozoites were also observed in crisis form outside erythrocytes (B3). At the 0.52 mg/ml dose) crisis forms with enlarged, thickened, compacted chromatin (C1), and cytoplasmic loss (C3) were seen. Schizonts and trophozoites appeared to have irregular shapes (C2, C4), but some were remained in good condition. At a concentration of 2.6 mg/ml schizonts failed to grow and looked damaged. Nuclei were found at the periphery of the parasite cytoplasm, and the chromatin was thick, compact, and dark (D1). Many trophozoites appeared in crisis form, also marginalized to the erythrocytes edges, and were more pycnotic (D2, D3, and D4). Parasite density was clearly lowered. At the highest extract dose (13 mg/ml) almost all parasites appeared in crisis form, schizonts were damaged, and failed to growth (E1). Some crisis forms were also observed outside erythrocyte (E2) and most erythrocytes were free from parasites (E3, E4).

### The Degree of Parasite and DNA Parasite Density

**Fig. 2:**
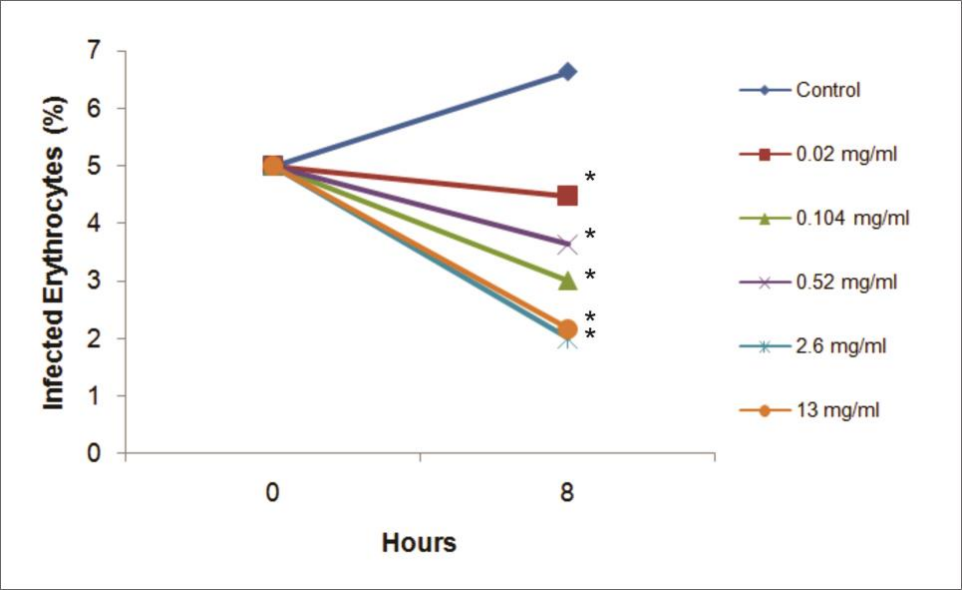
Graph of degree of parasite. Graph was the degree of parasite comparison between 0 and 8 hours. Star sign (*) showed significant difference compared to control (*P*<0.05)

Number of parasites from asynchronous culture was calculated by calculating degree of parasite-infected erythrocytes under the microscope. One-way ANOVA test of the degree of parasite showed a significant difference among groups with *P* =0.004 (Fig. 2). All of treatment groups yielded significantly decreased degrees of parasite-infected cells compared to control (*P* <0.05). Moreover, at the highest concentrations (2.6 mg/ml and 13 mg/ml) the extract significantly reduced the degree of parasite further as compared to cells exposed to the lowest concentration of the extract (*P*<0.05). Pearson correlation test showed there was significant inverse correlation between crude extract doses and the degree of parasite (r = −0.772, *P* = 0.000).

The percentage inhibition of secondary metabolite extract on the percentage of infected erythrocytes was obtained through the following formula: % inhibition= (% parasite positive control - % parasite treatment) / % parasite positive control) ×100. The degree of parasite inhibition percentage for 8 hours of treatment incubation showed that dose 2.6 mg/ml and 13 mg/ml had the highest effect with 69.8 % and 67.3% inhibition, respectively. ID50 (dose of 50% inhibition) was determined by probit analysis followed by linear regression analysis. This probit analysis compared log-doses and probit score converted from inhibition percentage. The test results obtained from the linear regression equation Y = 0.3 X+5.182 with R^2^=0.727. ID50 of the equation was obtained by 0.23 mg/ml in parasite culture.

**Fig. 3:**
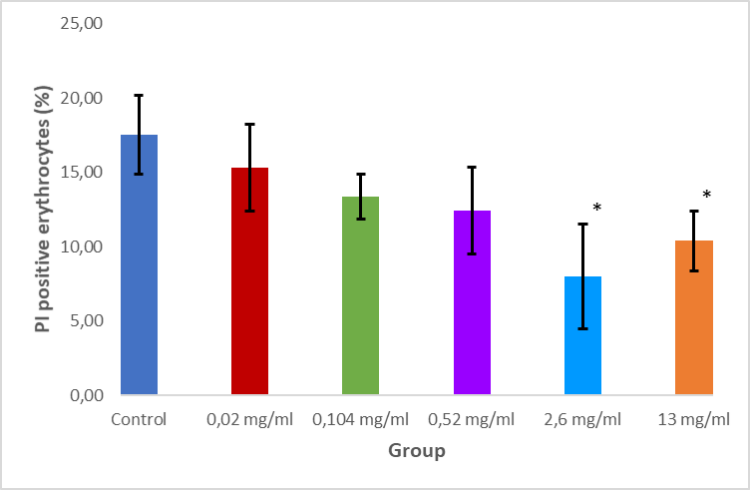
Graph of DNA parasite density stained by Propidium Iodine (PI) using flow cytometry. Mean of flow cytometry M2 gate at 8 hours for each group was shown in bar graph. Star sign (*) showed significant difference compared to control (*P*<0.05)

The effect of the extract on the parasites was also measured by determining the fraction of erythrocytes containing DNA using flow cytometry (Fig. 3). DNA parasite density was decreased significantly only at doses of 2.6 mg/ml and higher. However, there was a significant inverse correlation between crude extract doses and DNA parasite density (r =−0.753; *P* =0.000) which according to Dancey and Reidy's (2004) categorization included in strong correlation.

## Discussion

According to our previous study (13), crude metabolite extract *S. hygroscopicus* subsp. hygroscopicus that contained eponemycin, is a potential candidate for new malarial drug by inhibiting UPS function of the parasite and cause stress and dead of *P. berghei* in vivo (13). This previous result was similar to this study. This extract could cause morphological damage, decrease percentage of infected erythrocytes, and DNA parasite density. It has demonstrated antimalarial activity of *S. hygroscpicus* subsp. hygroscopicus crude metabolite extracts on UPS system of *P. falciparum* 3D7 in vitro. UPS as a system that consist of ubiquitin and multi-subunit protease complex in all eukaryotic cells generally has central role for quality control of gene expression, and response toward oxidative stress. The *Plasmodium* UPS shows low similarity to the ubiquitin proteasome system in humans and this fact increases the importance of UPS as an unique target for studies in malaria challenging for therapeutic molecules development (17).

The synergism of tagging and degradation of misfolded protein that are no longer needed has ensure the changes of parasites morphological state that are crucial for pathogenicity and infectious cycle which now has been targeted for effective drug discovery and synthesis (18–20). Proteasome of *Plasmodium* has been shown its existence indirectly by using its inhibitor. First documented is the effect of proteasome inhibitor salinosporamide A on in vitro and in vivo parasite development. Salinosporamide A (NPI-0052) still need to be further explored to evaluate the safety profile for its use against malaria (15). Another proteasome inhibitor is lactacystin which can inhibit exoerythrocytic and erythrocytic stages but not sporozoite of *P. berghei*. But, lactacystin has low therapeutic index which can clear rat *Plasmodium* infection, but none of treated rats survived (21). Subsequent reports document sensitivity to another proteasome inhibitor: MLN-273, MG132, MG115, Z-L3-VS, Ada-Ahx3-L3-VS, bortezomib, gliotoxin, and the greatest sensitivity being exhibited towards epoxomicin, one of epoxyketone group. Compared to lactacystin treatment, eponemycin treatment can maintain mice survival with no crisis parasites survived and multiplied (13).

Based on the results of this in vitro study, analog eponemycin showed effectively disrupts the development of parasite stages. This study result showed that the polyubiquitinated protein accumulation in the parasite lysate increases with increasing dose of eponemycin analog on *S. hygroscopicus* metabolites extract causing inhibition of *P. falciparum* morphological progression proved by parasite DNA analysis using flow cytometry and parasite microscopic analysis. The parasite determination on the two methods flow cytometry and optical microscopy were almost similar (16).

Eponemycin and its analog are one of natural peptide in epoxyketonegroup which is used in this research. Until date, peptide *α'β'-epoxyketone* is known as the most specific and potent natural from the first generation of proteasome inhibitors (22). Epoximycin and dihydroponemycin (eponemycin synthetic analog) share an α',β'-epoxyketone pharmacophore and linear peptide backbone, but they have some differences in their left-, central-, and right-hand fragments. These differences contribute to their binding specificity, inhibition kinetics, and anti-proliferative activities (23). Epoxomycin most potently inhibits CT-L activity of 20S proteasome, while dihydroeponemycin inhibits CT-L and caspase-like sites with similar rates. They are specific for inhibiting proteasome only and do not inhibit other non-proteosomal proteases (24).

Epoxyketone could react with amino and hydroxyl groups of proteasome N-Terminal threonine with its warhead deactivate the proteasome (19). This mechanism based on two-step reaction, the attack of carbonyl group of epoxyketone moiety by catalytic hydroxyl followed by the opening of the epoxy ring by the free α-amino group to form a cyclical morpholino ring (24). This interaction forms two covalent bonds, making the inhibition irreversible and selective (25). Eponemycin specifically binds to proteasome despite of its common peptide backbone that similar to serine/cysteine protease inhibitors and inactivate the catalytic site of proteasome (26).

Widely known proteasome inhibition at catalytic site leads to cell cycle arrest and activation of apoptotic pathway. This exact mechanism likely occurs to parasites proteasome which its inhibition blocks the cell cycle progression, consistent with our findings that with compound doses of 2,6 mg/ml and 13 mg/ml significantly increased the number of apoptotic cell in vitro. Our previous study also observed similar effect with eponemycin analog from *S. hygroscopicus* metabolite extract that halt the progression of *Plasmodium* morphological development and reduced parasite levels *in vivo* (13). It stated in trophozoite and schizont stages of *P. falciparum*, the proteasome subunit β5 of core proteasome shown to be present in the cytoplasm and nucleus of blood stage parasites. Considering the rapid DNA replication during erythrocytic trophozoite stage where there is distinct up-regulation in the expression in the mid/late trophozoite to early schizont stages coinciding with the active replication window (27). Transcriptome analysis has also shown that during late trophozoite to schizont stage transformation, there is up-regulation of ubiquitin – proteosomal degradation genes signed its importance pathway in *Plasmodium* (20). Therefore, UPS is potential target site of antimalarial therapy.

In this study, we could prove that crude metabolite extract of *S. hygroscopicus* subsp. hygroscopicus is a potential antimalarial agent which caused morphological damage, decrease the percentage of infected erythrocytes, and DNA density of *P. falciparum* 3D7.

## Conclusion

The metabolite extract affected the morphology of almost all of parasite asexual stages. Schizonts and trophozoites failed to grow and appeared damaged with pycnotic cores and loss of cytoplasmic content. At 8 hours there was a significant decrease in DNA parasite density in culture exposed to 2.6 mg/ml and 13 mg/ml of the extract. The degree of parasite-infected erythrocytes decreased when exposed to > 0.02 mg/ml of the extract. Metabolite extract of *S. hygroscopicus* subsp. hygroscopicus causes morphological damage, decreases the degree of parasite-infected erythrocytes and the DNA density of *P. falciparum* 3D7 in vitro.
